# Reduction Dynamics of Doped Ceria, Nickel Oxide, and Cermet Composites Probed Using In Situ Raman Spectroscopy

**DOI:** 10.1002/advs.201500146

**Published:** 2015-09-25

**Authors:** Robert C. Maher, Paul R. Shearing, Edward Brightman, Dan J. L. Brett, Nigel P. Brandon, Lesley F. Cohen

**Affiliations:** ^1^ The Blackett Laboratory, Imperial College LondonPrince Consort RoadLondonSW7 2BZUK; ^2^ The Electrochemical Innovation LabDepartment of Chemical EngineeringUniversity College LondonTorrington PlaceLondonWC1E 7JEUK; ^3^ National Physical LaboratoryHampton RoadTeddingtonMiddlesexTW11 0LWUK; ^4^ Department of Earth Science and EngineeringImperial College LondonLondonSW7 2BPUK

**Keywords:** catalysis, ceria, fuel cells, Raman spectroscopy, reduction

## Abstract

The redox properties of gadolinium doped ceria (CGO) and nickel oxide (NiO) composite cermets underpin the operation of solid oxide electrochemical cells. Although these systems have been widely studied, a full comprehension of the reaction dynamics at the interface of these materials is lacking. Here, in situ Raman spectroscopic monitoring of the redox cycle is used to investigate the interplay between the dynamic and competing processes of hydrogen spillover and water dissociation on the doped ceria surface. In order to elucidate these mechanisms, the redox process in pure CGO and NiO is studied when exposed to wet and dry hydrogen and is compared to the cermet behavior. In dry hydrogen, CGO reduces relatively rapidly via a series of intermediate phases, while NiO reduces via a single‐step process. In wet reducing atmospheres, however, the oxidation state of pure CGO is initially stabilized due to the dissociation of water by reduced Ce(III) and subsequent incorporation of oxygen into the structure. In the reduction process involving the composite cermet, the close proximity of the NiO improves the efficiency and speed of the composite reduction process. Although NiO is already incorporated into working cells, these observations suggest direct routes to further improve cell performance.

## 1 Introduction

The behavior of doped ceria, nickel oxide (NiO), and their cermet composite surfaces under reducing conditions is of significant interest for a wide range of catalytic applications.^1^ Ceria is of particular interest for solar production of carbon monoxide and hydrogen,^2^, ^3^ as an electrolyte for solid oxide fuel and electrolysis cells (SOFCs and SOECs),^4^, ^5^, ^6^ and as a catalyst and support for catalytic converters.^7^ Nickel, which is often produced from NiO precursors, is widely used as a catalyst for a variety of chemical reactions such as the hydrogenation of unsaturated compounds,^8^ production of carbon nanotubes,^9^ oligomerization,^10^ and polymerization.^11^ Cermet composites of Ni and ceria are also commonly used as electrodes within SOFCs and SOECs.^6^, ^12^, ^13^ As a result, the characteristics of the reduction of NiO, doped ceria, and their cermet composites are of significant interest.

Ceria is partially reduced from Ce(+IV) to Ce(+III) when exposed to high temperatures or reducing atmospheres^13^

(1) $${\mathrm{CeO}}_{2}+xH_{2}={\mathrm{CeO}}_{2-x}+xH_{2}O$$

The reduction of ceria exposed to hydrogen is an endothermic reaction^14^ known to be strongly affected by a number of factors, including the doping,^15^ specific surface area,^16^ crystallinity,^17^ and the presence of metals.^17^, ^18^ While pure ceria is of significant interest in its own right, the ionic conductivity is typically maximized through the addition of a suitable aliovalent dopant such as samarium and gadolinium.^4^, ^19^ Such dopants generate oxygen vacancies, increasing oxygen ion conduction and as a result they have a significant effect on the redox properties. The partial reduction of ceria‐based electrolytes can become an issue during the operation of SOFC and SOEC systems, as mixed ion electrical conduction develops resulting in an internal short circuit and degrade cell performance.^20^

Partially reduced ceria is readily reoxidized in the presence of oxygen, either a source of O_2_ or when exposed to oxygen‐containing molecules such as carbon dioxide or water vapor.^17^, ^21^ In the case of water vapor, the reoxidation process is driven by the dissociation of water molecules absorbed onto the ceria surface and subsequent incorporation of the resulting surface oxygen ion species into the ceria accompanied by the evolution of hydrogen.^17^ This process is shown schematically in Figure S1 (Supporting Information). In recent years, the thermochemical redox cycling of ceria has been proposed as an efficient means of producing CO and H_2_ from carbon dioxide and water, respectively, using concentrated solar thermal energy.^2^

NiO reduction is a key issue for SOEC and SOFC systems, where the reduction and oxidation (redox) tolerance of Ni‐based electrodes is an important practical consideration.^23^ Such processes have been studied in detail for SOFC anodes, the results of which are directly applicable to SOECs. Porous electrodes with high surface areas are typically produced through the in situ reduction of NiO‐electrolyte cermet.^22^, ^23^ However, dimensional changes induced by subsequent redox cycles can lead to a gradual degradation in performance via a reduction in surface area and the eventual catastrophic failure through component cracking and delamination.^24^ A high redox durability therefore greatly enhances the lifetime, stability, and operation of SOFCs. Although the redox mechanism in SOFC anodes has been studied widely using techniques such as thermogravimetry,^25^ secondary electron microscopy (SEM),^26^ electrical conductivity measurement,^27^ X‐ray diffraction (XRD),^27^ and X‐ray tomography,^28^ these methods are not generally restricted to the active surface layers and moreover lack real‐time dynamic information.

Raman spectroscopy^29^ is an ideal tool for the in situ study of chemical processes occurring at active surfaces.^30^, ^31^, ^32^ We employ it here to investigate the reduction dynamics of gadolinium doped ceria (CGO), NiO, and NiO‐CGO cermet surfaces exposed to both dry and wet reducing environments as a function of temperature. We show that the presence of NiO in the composite cermet gives rise to a number of competing chemical processes such as the dissociation of water absorbed onto the surface and hydrogen spillover from metallic Ni in close proximity, which can have a dramatic effect on the reduction process.

## 2 Results and Discussion

**Figure 1:**
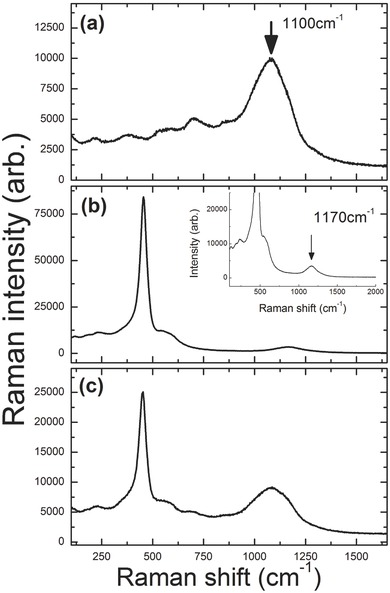
Extended Raman spectra of a) NiO, b) CGO, and c) NiO‐CGO cermet taken at 600 °C in flowing nitrogen using a 514 nm laser. Inset to (b) shows the Raman spectrum obtained from CGO at 600 °C emphasizing a small second‐order Raman mode at 1170 cm^−1^ close to the position of the principle NiO mode. See text for further details.

**Figure** **1**a–c shows typical Raman spectra of NiO, CGO, and NiO‐CGO cermet samples taken in situ at 600 °C. These spectra were collected at high temperature to ensure that the contribution of any remaining organic contaminants is minimized. Details of the sample preparation and of the conditions of all measurements are given in the Experimental Section.

The spectral response from pure NiO is shown in Figure 1a and is characterized by a single, broad, and intense peak at ≈1100 cm^−1^ arising from the 2LO 2 phonon scattering mode.^33^ The CGO Raman spectrum, shown in Figure 1b, is dominated by a single, sharp, and intense mode at 460 cm^−1^ which is associated with the *F*_2*g*_ symmetric breathing mode of the oxygen atoms around the Ce cations in the CGO.^34^ Several smaller modes are also visible although only the second‐order mode seen at 1170 cm^−1^, as shown in the inset to Figure 1b, which is a result of the mixing of the *A*_1*g*_, *E_g_*, and *F*_2*g*_ modes of CeO_2_, is of interest in this case.^35^ This is close to, and in fact overlaps with, the broad NiO peak. As a result, in some instances, careful deconvolution of these two peaks is required in order to correctly analyze and interpret the data. The spectral response of the fully oxidized NiO‐CGO cermet is thus a direct combination of these two spectra, as shown in Figure 1c, where the relative intensities of the individual features are directly related to their intrinsic Raman cross‐section and concentration. In this case, while the triply degenerate 460 cm^−1^ mode of CGO dominates the spectrum, owing to its large Raman cross‐section, the NiO mode is still relatively intense. As a result, the features of both CGO at 460 cm^−1^ and NiO at 1100 cm^−1^ can be monitored together in order to characterize the average oxidation state of both components in real time, as will be demonstrated.

Observed Raman mode intensities typically depend on the intrinsic Raman cross‐section of the analyte, its local constituent concentration within the sampling volume, and to a lesser extent on the surface roughness and resultant scattering conditions. The inevitable variation in these parameters across complex surfaces often leads to a slight variation in signal. However, monitoring a single location removes any contribution from the variation in surface roughness to the signal and allows oxidation states to be quantitatively determined with respect to their initial state. Peak intensities extracted from the monitoring measurements were normalized to the intensity of the initial spectrum obtained from the fully oxidized surface. This removes the effect of intensity variations over different measurements due to local scattering conditions and allows the oxidation state of each sample to be directly compared. As the samples are reduced, the Raman intensity decreases in direct response to the decrease in oxygen content of the sample and the resulting changes in the optical properties of the surface, such as absorption and reflection. As a result, the Raman response of each sample is a direct indication of its oxidation state.^31^

**Figure 2:**
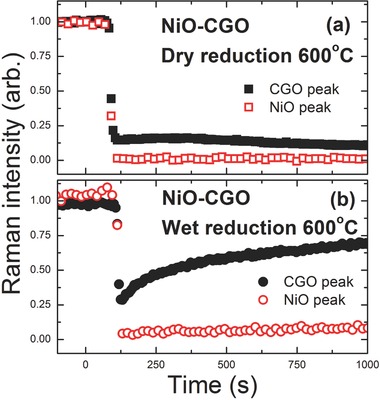
Normalized intensities of the NiO and CGO Raman peaks monitored as a function of time from a NiO‐CGO composite exposed to a) dry and b) wet 75% N_2_:25% H_2_ flowing at 100 cm^3^ min^−1^. Reduction of NiO and CGO appears to be stronglly coupled in both cases, although the oxidation state of the CGO surface strongly recovers for the cermet exposed to the wet atmosphere. See text for further details.

The reduction dynamics of the CGO‐NiO composite were characterized by monitoring the intensities of their respective Raman modes from a single location while it was exposed to both dry and wet hydrogen for temperatures between 400 and 700 °C in 50 °C intervals. **Figure** **2**a shows the normalized intensities of the NiO and CGO Raman modes monitored from the surface of the NiO‐CGO composite exposed to a dry reducing atmosphere at 600 °C. Spectra were collected continuously from the samples during both the purge and reducing phases of the measurement to ensure that the oxidation state was fully characterized throughout the measurement.

The reduction of both the CGO and NiO components of the composite appears to proceed via a single‐step process 70 s after initial exposure to the reducing environment, which indicates that they could be coupled. The NiO peak intensity then reduces to zero within 100 s of exposure. However, the CGO surface is only partially reduced. The CGO peak never reduces to zero and in fact recovers very slightly ≈250 s after exposure before eventually stabilizing to about 10% of its initial intensity.

Figure 2b shows the normalized intensities of the NiO and CGO Raman peaks monitored from a CGO‐NiO cermet composite exposed to wet reducing atmosphere at 600 °C. Again the intensities of both the CGO and NiO peaks decrease rapidly together around 70 s after the surface exposed to the reducing environment, suggesting that the reduction of the components is coupled. The NiO peak behaves similarly to the case when NiO is exposed to dry hydrogen, reducing to zero within 100 s of exposure. However, the reduction dynamics of the CGO component are strikingly different. Initial CGO reduction is strongly correlated with the NiO component. However, the Raman intensity of the CGO component does not drop below 25% of its initial intensity, and then shows significant recovery to ≈70% of its original intensity 15 min after exposure to the reducing environment. Clearly the reduction dynamics are significantly different depending on whether the reducing environment is humidified or not. As well as this, the reduction dynamics were found to be broadly similar at the other temperatures. The integrated intensities of the CGO and NiO components of cermets reduced in humidified reducing atmospheres at 500, 650, and 700 °C are shown in Figure S2 (Supporting Information) demonstrating this. By comparing the reduction behavior of CGO‐NiO at different temperatures it is clear that the dynamics of the surface reduction of CGO‐NiO in humidified hydrogen are broadly similar in that the CGO is significantly reduced before recovering. However, by it is also clear that the recovery equilibrium state of the CGO component varies with temperature from about 45% of the original intensity at 500 °C, 70% at 600 °C, 75% at 650 °C, and 87% at 700 °C.

**Figure 3:**
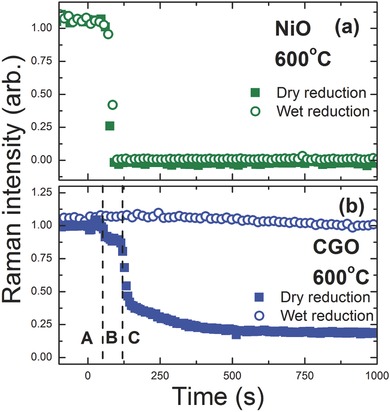
Normalized intensities of the a) NiO and b) CGO Raman peaks monitored as a function of time from pure NiO and CGO samples exposed to dry and wet 75% N_2_:25% H_2_ flowing at 100 cm^3^ min^−1^. The reduction of NiO is unchanged by the presence of water in the reduction gas, while the CGO appears to be strongly stabilized. See text for further details.

In order to investigate these differences further, the reduction dynamics of pure samples of the NiO and CGO component materials under the same conditions were investigated. **Figure** **3**a,b shows the normalized integrated intensities of the NiO and CGO Raman modes, monitored as a function of time from pure samples exposed to wet and dry 75% N_2_:25% H_2_ flowing at 100 cm^3^ min^−1^ at 600 °C. The reduction characteristics of NiO exposed to either dry or wet hydrogen are observed to be exactly the same, as shown in Figure 3a. The Raman intensity of the NiO mode drops rapidly ≈70 s after exposure to the reducing environment, becoming undetectable within 100 s of exposure. Indeed the reduction of NiO appears to proceed precisely as it did within the composite material. This suggests that the presence of either CGO or H_2_O has very little, if any, effect on the dynamics of NiO reduction.

In contrast, the dynamics of the reduction of CGO are highly sensitive to the humidity of the reducing environment as well as being different to the dynamics of the CGO component in the CGO‐NiO composite material. Reduction of pure CGO exposed to dry hydrogen is observed to proceed in distinct stages related to the formation of various phases, as shown in Figure 3b. Initially there is a clear drop in the intensity of the Raman mode upon initial exposure of the dry hydrogen to the surface. The peak immediately stabilizes for ≈30 s before rapidly decreasing to around 40% of its initial intensity. This rapid decrease in intensity is then followed by a much more gradual decrease with the signal stabilizing at around 20% of its initial intensity ≈500 s after hydrogen exposure. These regions can be related to the ceria stoichiometry with the initial form of CeO_2_ reduced to CeO_1.85_ and finally CeO_1.72_. 31 However, when exposed to wet H_2_, the reduction dynamics for CGO are changed to such an extent that the sample's surface is almost completely stabilized over the same timescale. In fact there is a small increase in the intensity of the CGO peak which results from an increase in the concentration of oxygen ions at the CGO surface driven by the presence of significant amounts of hydrogen. As more hydrogen is absorbed onto the surface the chemical potential draws more oxygen from the CGO subsurface to the near surface where it is then detected as an increase in the Raman intensity. This initial increase in the CGO Raman intensity is generally not observed for the NiO‐CGO cermet because the rate of reduction is increased so substantially.

The key question is: how can a simple humidification of the reducing gases be responsible for such a radical change? The difference in the evolution of the CGO Raman intensity for surfaces exposed to dry and wet H_2_ is a direct result of the physical interactions occurring on the surfaces. When exposed to dry H_2_, the CGO surface is partially reduced from Ce(+IV) to Ce(+III) releasing H_2_O. As the oxygen content of the upper CGO surface decreases, the Raman intensity decreases correspondingly. In the case of wet H_2_, the Ce(+III) formed during the initial reduction process drives the rapid reoxidation of the surface through H_2_O dissociation accompanied by H_2_ release and subsequent oxygen incorporation into the crystal structure. The chemical composition of the CGO surface is almost completely stabilized by this process because, under these conditions, this exothermic process is more energetically favorable than the surface reduction and proceeds at a much higher rate.

This is in striking contrast to the dynamics involved during the reduction of the CGO component of the CGO‐NiO under either dry or humidified reducing conditions. Exposed to a dry reducing environment, the CGO component appears to reduce faster compared to pure CGO under the same conditions, with the rapid decrease in the CGO Raman peak being coincident with that of the NiO peak, without intermediate steps. Not only is the reduction quicker, the CGO surface also appears to be more strongly reduced, with the intensity of the CGO peak dropping to about 10% of its initial intensity compared to 18.5% for the pure CGO after 15 min exposure to the reducing environment. When exposed to a humidified reducing atmosphere, the CGO component of the composite surface does not appear to be stabilized against reduction by the presence of water vapor in the environment, as is the case for pure CGO.

**Figure 4:**
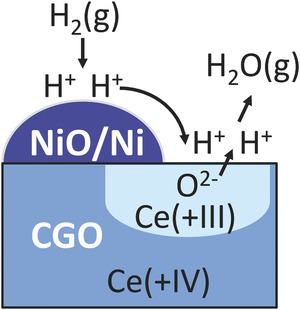
Schematic illustration of the hydrogen spillover process. Gaseous hydrogen is absorbed and dissociates on the NiO/Ni (dark blue area) surface near the interface with CGO (dark gray area). The absorbed hydrogen ions migrate to the CGO where they react with an oxygen ion from the CGO subsurface forming H_2_O and oxygen vacancies within the CGO. The H_2_O then desorbs from the surface into the gas phase. Eventually a significant region of reduced Ce(+III) (light blue area) is formed in close proximity to the NiO/Ni‐CGO interface. See text for further details.

These observations clearly show that the reduction dynamics of the CGO component of the composite are significantly affected by the presence of the NiO. This can be directly attributed to the effect of hydrogen spillover from the reducing NiO, a process that is schematically illustrated in **Figure** **4**. The process of hydrogen spillover increases the rate of reduction and reduces the stability of the affected CGO. Such effects have been previously shown to result in the significant reduction of ceria‐based ceramics that are in close contact with Ni/NiO.^18^ In a similar way, the increase in the effective hydrogen concentration on the CGO surface induces the formation of a strongly reduced surface layer of CGO in close contact with the NiO/Ni component, resulting in the decreased Raman intensity. The slight recovery in the CGO peak intensity observed ≈250 s after sample exposure is attributed to the transport of oxygen ions through the CGO matrix to equilibrate the chemical potential with the surface. This is a separate process from the dissociation of water by Ce(III) followed by oxygen incorporation into the CGO surface. Both of these processes result in a recovery of the oxidation state of the CGO surface although oxygen ion migration through the CGO is much less effective at the temperatures studied here.

This insight suggests that the effect of the initial increase in effective hydrogen concentration experienced by the CGO in close proximity to the interface with the NiO/Ni component driven by hydrogen spillover is stronger than that of H_2_O dissociation and subsequent oxygen incorporation. As a result, a significant proportion of the CGO surface in close proximity to the NiO/Ni is reduced in close correlation to the NiO reduction. It is only after this has occurred and sufficient Ce(+III) is formed at the surface, that the processes of H_2_O dissociation and oxygen incorporation into the CGO can occur, resulting in the recovery of the CGO Raman intensity as the surface becomes partially reoxidized.

Of course, reduction of the CGO surface exposed to dry hydrogen will result in the evolution of water which might be expected to then interact with the surface at reduced sites resulting in a significant reoxidation of the surface. However, it is clear from our results that the amount of H_2_O produced during the surface reduction process and its residence time on the surface is insufficient for significant interaction to occur.

The behavior of mixed metal/CGO cermet composites under reducing conditions is an important consideration for a number of applications, particularly for SOFCs operating in the intermediate temperature range where CGO is a popular electrolyte.^36^ From these observations it is clear that the reduction dynamics of the surfaces of ceria‐based ceramics are highly sensitive to the specific composition of supplied gas and their environment. However, it is interesting to ask what the implication of these effects might be over longer timescales?

**Figure 5:**
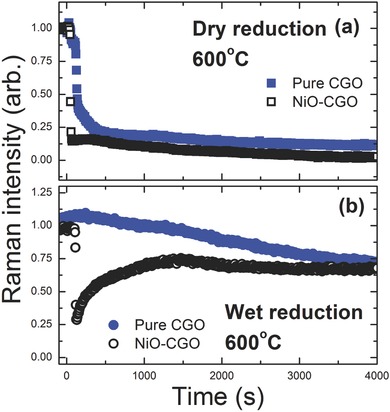
Normalized intensities of the CGO peak monitored from pure and NiO‐CGO composites under a) dry and b) wet 75% N_2_:25% H_2_ flowing at 100 cm^3^ min^−1^ over long timescales. CGO within the cermet appears to reduce more rapidly, and without intermediate phases when exposed to dry H_2_. It is also more strongly reduced. When exposed to wet H_2_, the CGO within the composite is reduced relatively quickly before recovering on longer timescales, tending to the same oxidation state observed for pure CGO. See text for further details.

**Figure** **5** shows the normalized intensity of the CGO Raman peak for pure CGO compared to that of CGO obtained from the CGO‐NiO cermet exposed to (a) dry and (b) wet reducing environments for more than an hour. In the case of the dry reducing environment, the rate of the dynamics of the CGO reduction is significantly increased when CGO is in a mixed NiO/Ni cermet composite, as previously seen. On longer timescales a very slight recovery in the oxidation level of the CGO surface similar to that observed for the NiO‐CGO cermet occurs in the pure CGO ≈20 min after exposure to the reducing environment. After this point there is a gradual and continuous decrease in the CGO Raman peak intensity which stabilizes around 10% and 2% of the original peak intensity for the pure CGO and CGO component of the NiO‐CGO cermet, respectively. It is clear then that the surface of the CGO component of the NiO‐CGO is reduced to a greater degree than that observed for the pure CGO.

In wet reducing environments, Figure 5b shows that the long‐term behavior of the CGO surface in response to the reducing environment for pure CGO (same data are shown in figure 3b on a different timescale) and the component CGO of the NiO‐CGO cermet is essentially the same. This longer term monitoring of the pure CGO surface shows that the oxidation state is not completely stabilized by the presence of water vapor in the fuel stream, instead decreasing gradually. More than an hour after the surfaces were first exposed to the wet reducing environment at 600 °C, the CGO Raman intensity stabilizes at 72% and 67% of the initial intensity for the pure and composite CGO, respectively. It is only during the initial interactions that the effect of the NiO/Ni component of the composite is observed. The recovery in the CGO Raman peak intensity in the composite is observed to peak around 1350 s after the surface was first exposed to the reducing environment. This peak in intensity is followed by a slight decrease before the surface stabilizes around 2200 s. As noted earlier, the recovery state of the CGO component varies with temperature from about 45% of the original intensity at 500 °C, 70% at 600 °C, 75% at 650 °C, and 87% at 700 °C. This is due to the increasing oxygen ion conductivity of the CGO as the temperature is increased.^4^ At 500 °C the oxygen from water dissociated at the surface does not penetrate into the surface quickly enough before reacting with hydrogen to oxidize the surface more than to 45%. At higher temperatures, 700 °C for example, the oxygen released from water dissociated at the surface is absorbed into the surface more quickly as the oxygen ion conductivity is higher, and as a result more of the reduced CGO surface is reoxidized. As a result the catalytic activity of these materials in humid reducing environments can also be expected to vary with temperature given the variation in the extent of the CGO reduction.

Our observations quantify how the reduction dynamics of the CGO electrolyte change when mixed with NiO in the cermet. We can attribute this to the direct effect of differences in the dynamics of the key chemical processes occurring at the interfaces between the constituent materials—namely, the effect of hydrogen spillover from the NiO/Ni to the contacted CGO that we monitor directly. While, previous studies have shown that CGO should be thermodynamically resistant to reduction below 800 °C,^4^ the thermodynamics of CGO reduction are modified with sintering temperature.^37^ Studies have shown that the CGO component of cermet matrices can be reduced at temperatures below 800 °C as hydrogen and oxygen are transferred from and to metals that are in direct contact with the CGO via spillover reactions.^38^ These observations are consistent with our results, suggesting that the NiO/Ni promotes CGO reduction. Dissolution of Ni into CGO is another potentially complicating factor. Studies have shown that Ni from NiO can dissolve into ceria even at relatively low temperatures such as 400 °C,^39^ reducing the sharpness of the interface between the Ni/NiO and CGO. This might reduce the effectiveness of the hydrogen spillover process. The dissolution of Ni into the ceria lattice would be expected to increase over time and be greatly accelerated by repeated redox cycling. In our case we can assume that the effect of the dissolution of Ni into the CGO is minimal given that we have minimized the sintering time of our samples and limited our investigation to a single‐reduction step. Further investigation of repeated redox cycling would be required to clarify the effect of Ni dissolution on the reduction of CGO.

The reduction and oxidation dynamics of nickel surfaces are of considerable interest as nickel is an important catalyst, not only for fuel cells but also for many other applications. Despite this, there is considerable debate within the literature as to the details of the kinetics of NiO reduction and a wide range of activation energies (*E*_a_) have been reported. Richardson et al. have reviewed the *E*_a_ values found in the literature and found that they ranged between 17 and 133 kJ mol^−1^ and concluded that the measured value of *E*_a_ was dependent on the type of sample and its preparation.^40^

It is generally agreed that the reduction process in bulk NiO begins with the chemisorption of hydrogen onto the surface. This is followed by an induction period which proceeds at a minimal reaction rate, with an associated initial time during which NiO at the surface is first converted to Ni.^25^ Such surface dynamics are important since electrode percolation and therefore conductivity will only occur when a “shell” of connective Ni is formed. Therefore this should also be an indication of when electrical conductivity is reached. These surface interactions are of particular importance for catalytic processes given their surface sensitive nature.

Relating the different reaction mechanisms involved in the reduction process to the changes in the detected Raman intensity is challenging given the complexity of the surface reaction processes. However, we can assume that the time constant related to the disappearance of the NiO peak, as measured at different temperatures, will be determined by the rate limiting mechanisms. As the NiO reduction is clearly represented by a single‐step process in the Raman response from the surface, unlike that of CGO, it is possible to determine the kinetics related to this rate limiting step.

In this case, we can split the process into two regions, (i) the initial phase during which the Raman intensity is relatively constant and (ii) a second phase during which the NiO Raman intensity drops from full intensity to zero, with time constants *t*_1_ and *t*_2_, respectively, as shown in Figure S3 (Supporting Information). *t*_1_ is associated with the initial time taken for the formation of Ni in the surface layers and *t*_2_ is related to the reaction front proceeding from the surface into the subsurface layers. For these measurements, *t*_2_ is relatively short in comparison to the integration time used for the experiment and as a result cannot be accurately measured at higher temperatures. As a result we have used only *t*_1_, measured as a function of temperature, to determine the activation energy for the rate limiting step.

From this, the activation energy for the reduction of NiO, as extracted from the time‐dependent Raman data obtained at different temperatures, was found to be 35.9 ± 4.6 kJ mol^−1^. This activation energy lies in the lower range of previously reported activation energies measured for the reduction of NiO^38^ which is entirely reasonable given that it is related to surface‐specific NiO reduction.

## 3 Conclusion

The dynamics involved in the reduction of CGO, NiO, and NiO‐CGO cermet composite surfaces are of great interest for a wide range of catalytic applications. Raman spectroscopy has been used to investigate these processes at the surfaces of pure CGO and NiO as well as NiO‐CGO cermet composites under both dry and wet reducing atmospheres as a function of temperature.

By monitoring individual peaks associated with different chemical species within the cermet, we have been able to chart the coupled dynamic processes occurring between gas, catalyst, and oxygen ion conductor. The presence of NiO/Ni within the NiO‐CGO cermets during the reduction process is observed to greatly enhance the CGO reduction due to hydrogen spillover. This increases both the speed and extent to which the constituent CGO is reduced compared to pure CGO subject to the same environment which would normally reduce via a series of intermediate phases. In humidified reducing atmospheres, the oxidation state of the pure CGO surface is strongly stabilized due to the dissociation of water by reduced Ce(III) followed by the subsequent incorporation of the released oxygen into the CGO. However, significant reduction of CGO within cermet composites subjected to humidified atmospheres was observed to occur, although this is followed by the swift CGO reoxidation due to later water dissociation and oxygen incorporation.

NiO reduction is observed to proceed through a single‐step process that is unaffected by the water vapor content or the presence of CGO. This allowed the activation energy for the surface‐specific reduction of NiO to be extracted from the time‐dependent Raman data obtained at the different temperatures investigated. This activation energy was found to be 35.9 ± 4.6 kJ mol^−1^, which is consistent with previously reported values.

The complexity of the interactions involved during surface reduction within operational systems as revealed here points to a number of strategies to optimize fuel cell performance. Clearly it is desirable to maximize the NiO to cermet interface preferably by nanostructuring the catalyst to create large surface to volume ratio of catalyst. Secondly, uniform dispersion of metal at the fuel/catalyst interface is required to prevent agglomeration. Based on these observations, novel routes to nanostructuring active surfaces such as Ni ex‐solution^41^ and infiltration^42^ techniques look to be promising ways to direct a rational design process. This would aim to improve the performance of SOFCs systems via improved control of the positioning and interaction of material interfaces and an optimization of operational conditions to allow for the oxidation states of critical, active surfaces to be well controlled.

## 4 Experimental Section

Cylindrical pellets of NiO were prepared by uniaxially pressing NiO powder (fuelcellmaterials, Ohio) at 1 ton for 30 s. The pellets produced had a thickness of ≈1 mm and a diameter of ≈12 mm. All ceria samples were prepared with 10% Gd doped CGO. NiO‐CGO cermet samples were prepared using an ink of NiO‐CGO powder with a composition of 60% NiO–40% CGO by weight (fuelcellmaterials, Ohio). The ink was prepared by mixing this powder with an ink vehicle and triple roll milling. The ink was then screen printed onto a YSZ (yttria‐stabilized zirconia) electrolyte support and fired at 1350 °C for 1 h (ramped at 5 °C min^−1^). Samples were then broken into smaller fragments in order to fit into the sample stage.

Sample surfaces were extensively characterized before and after reduction using a range of techniques. The surface roughness of the samples was characterized using a Zygo NewView 7000 Series 3D optical surface profiler. The root mean squared (RMS) roughness of each sample was measured using a 360 × 260 μm field of view. SEM images of the samples were also obtained using an FEI FIB 200 ion beam microscope, at 30 kV with 50 pA ion beam current used to generate the secondary electron signal. SEM images of the NiO and NiO‐CGO samples before and after reduction are shown in Figure S4 (Supporting Information). Elemental mapping of the samples was carried out using a JSM6400 scanning electron microscope. Images were collected at 30 kV with a 20 min collection time per map.

Raman measurements were made using a Renishaw RM‐2000 charge‐coupled device (CCD) spectrometer equipped with a 514 nm laser. Measurements were made with a ×50 long working distance objective resulting in a 1.5 μm diameter laser spot at the focal point. Spectra were taken with an incident intensity of 5 mW of laser power at the focal point and an integration time of 30 s. The laser power and integration time were optimized to provide a good signal‐to‐noise ratio, while minimizing laser heating of the sample. A high‐temperature catalytic stage from Linkam Scientific Instruments (UK) allowed measurements to be made from room temperature up to 1000 °C in a variety of reducing atmospheres. Raman spectra were obtained from the sample between room temperature and 700 °C, as reported by the stage controller.

Controlled flows of dry N_2_ and H_2_ gas were delivered to the sample in the stage through a system of calibrated mass flow controllers which allowed for gas flow rates ranging from 2 to 100 cm^3^ min^−1^. The N_2_ and H_2_ supply lines were combined after passing through the mass flow controllers. This gas flow could then be optionally humidified with 3% water vapor by passing through a humidifier, which consists of a bubble column situated inside a thermocirculator bath. All samples were reduced using dry 75% N_2_:25% H_2_ gas which was either used directly or after humidification flowing at 100 cm^3^ min^−1^.

Samples of compacted CGO, NiO, and NiO‐CGO cermet were reduced in situ with H_2_ between 400 and 700 °C in 50° increments. The system was purged before and after reduction of the sample surface by flowing N_2_ for 20 min over the sample. Raman spectra were continuously collected from the sample during the purging and reduction procedures to monitor the degree of oxidation of the sample surface. Spectral features were background corrected and fitted to mixed Gaussian and Lorentzian modes using Renishaw Wire 2 with further analysis performed using OriginLab 8.6. Integrated intensities were normalized to the initial intensity (at *t* = 0) to allow the different measurements to be directly compared.

The mass of each sample was measured ex situ before and after reduction to ensure that only the top surface of the samples was reduced. Samples were cooled in nitrogen post reduction in order to avoid any reoxidation of the sample. Table S1 (Supporting Information) shows the measured mass of the samples before and after reduction along with the change in mass as a percentage. The mass decreases by between 1.5% and 4.8% which is consistent with the partial or surface‐only reduction of the sample, compared to an expected change of ≈21% for full reduction.

## Supporting information

As a service to our authors and readers, this journal provides supporting information supplied by the authors. Such materials are peer reviewed and may be re‐organized for online delivery, but are not copy‐edited or typeset. Technical support issues arising from supporting information (other than missing files) should be addressed to the authors.

Supplementary
