# Supplementary material for: Reduction Dynamics of Doped Ceria, Nickel Oxide, and Cermet Composites Probed Using In Situ Raman Spectroscopy
Source: Adv Sci (Weinh). 2015 Sep 25;3(1):1500146. doi: 10.1002/advs.201500146 (PMC4989454; doi:10.1002/advs.201500146)
Supplement: Supplementary file 1 — Supplementary [file ADVS-3-0k-s001.pdf]

Copyright WILEY-VCH Verlag GmbH & Co. KGaA, 69469 Weinheim, Germany, 2015.

ADVANCED SCIENCE
Open Access

Supporting Information

for *Adv. Sci.*, DOI: 10.1002/advs. **201500146**

Reduction Dynamics of Doped Ceria, Nickel Oxide, and Cermet Composites Probed Using In Situ Raman Spectroscopy

*Robert C. Maher , * Paul R. Shearing , Edward Brightman , Dan J. L. Brett , Nigel P. Brandon , and Lesley F. Cohen*

Supporting Information

Title Reduction dynamics of doped ceria, nickel oxide and cermet composites probed using *in-situ* Raman spectroscopy

Robert C. Maher, Paul Shearing, Edward Brightman, Dan J. L. Brett, Nigel P. Brandon and Lesley F. Cohen*

Table S1. Mass of NiO samples before and after reduction. The average change in mass is only ~3% compared to the ~21% change expected for full reduction. This is consistent with partial reduction of the samples only.

T [C]	Mass before [mg]	Mass after [mg]	Mass change [%]
400	54.5	53.7	1.5
500	31.1	30.0	3.6
600	26.9	25.6	4.8
700	23.3	22.4	3.8

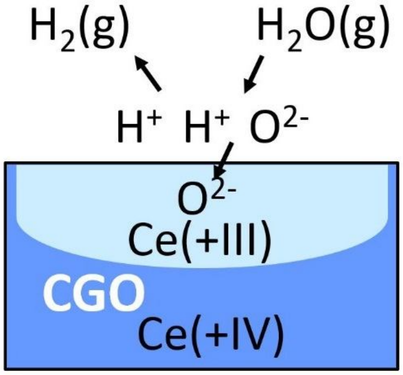

Supplemental figure 1: Schematic illustration of the process of water dissociation by Ce(III) and subsequent absorption of oxygen into the structure. Gaseous water is absorbed and dissociates on the reduced Ce(III) surface (light blue area). Oxygen is absorbed into the surface whilst the hydrogen evolves from the surface. See text for further details.

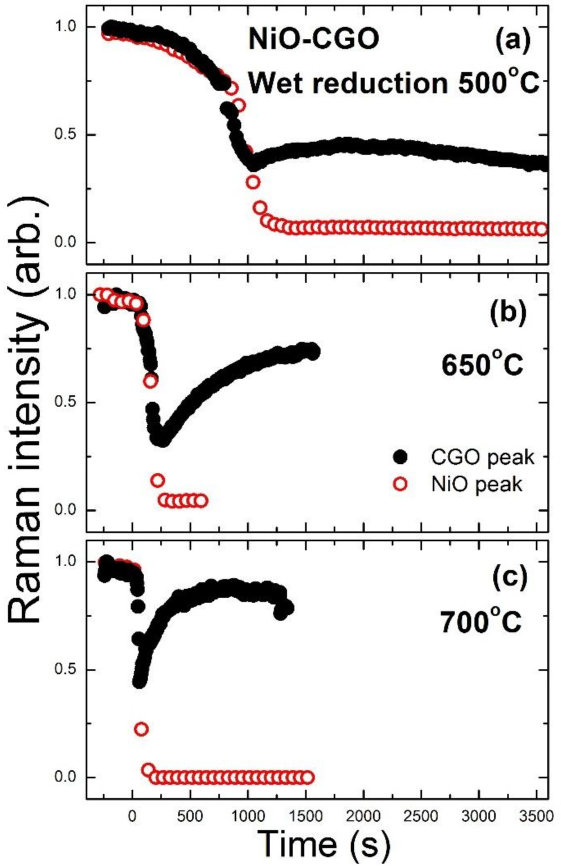

Supplemental figure 2: Normalised intensities of the NiO and CGO Raman peaks monitored as a function of time from NiO-CGO composites exposed to wet 75% N_2:25% H_2 flowing at 100 cm^3 min^{-1} at (a) 500, (b) 650 and (c) 700°C. Reduction of NiO and CGO are stronglly coupled while the CGO component recovers at all temperatures. See text for further details.

Assuming that the reduction of NiO proceeds as a single step process with Arrhenius-like behaviour, the time (t) taken for the complete reduction of surface oxide was determined by the $t = Ae\ –(E_a/k_BT)$. Where A is a constant, E_a is the activation energy of the reaction; k_B is the Boltzmann constant, and T is the temperature in Kelvin. This can be expressed as $ln(t) = lnA – E_a/k_BT$. This has been used to extract an activation energy of 35.9 ± 4.6 kJ mol^{-1} from the temperature depended reduction time data shown in figure S3.

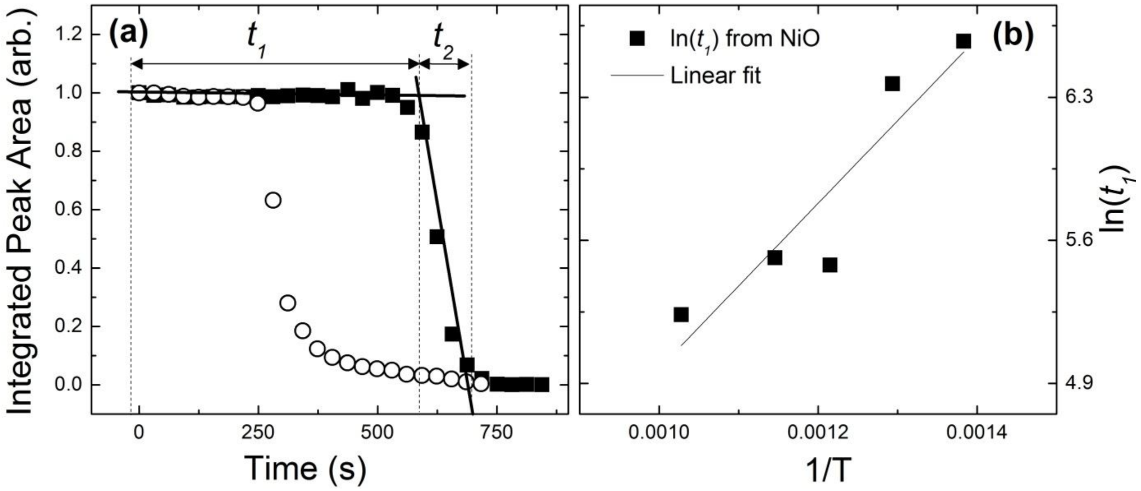

Supplemental figure 3: (a) Normalised integrated area of the NiO Raman peak from a pure NiO sample in a reducing environment as a function of time at (solid symbols) 500 and (open symbols) 600 °C. Samples were reduced in 75% N_2:25% H_2 gas flowing at 100 $cm^3\ min^{-1}$. Raman spectra were collected continuously using a 514 nm laser and an integration time of 30 s. (b) Natural logarithm of t_1 plotted against $1/T$ with the experimental data points (solid squares) fitted to $ln(Rate) = lnA - E_a/k_BT$ (solid line). The gradient of the fitted line allows the activation energy for the reduction of NiO to be calculated to be 35.9 ± 4.6 kJ mol^{-1}. See text for further details.

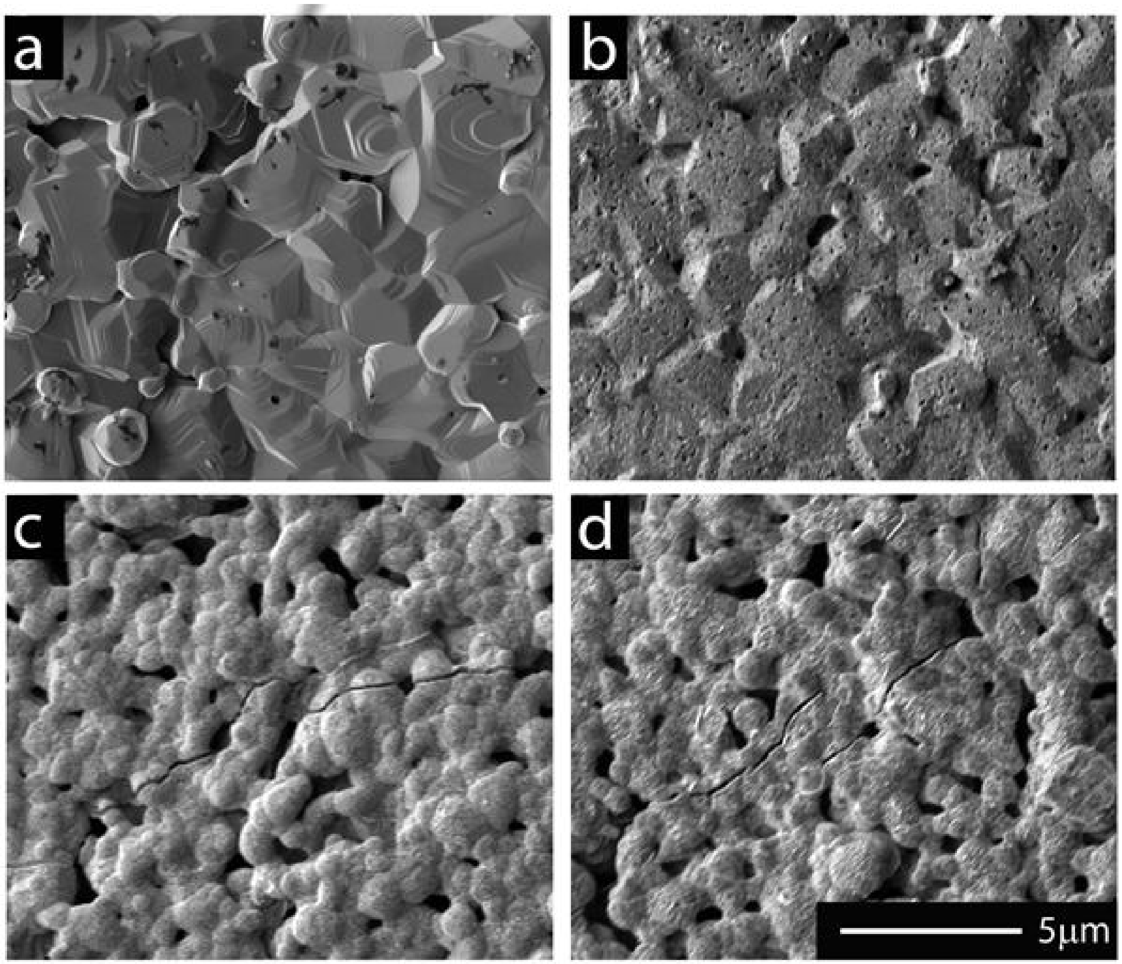

Supplemental figure 4: SEM images of the sample surfaces: NiO before (a) and after reduction (b), and the CGO-NiO cermet before (c) and after reduction (d). Scale bar appropriate for all images.
